# Comprehensive evaluation of fuel properties and complex regulation of intracellular transporters for high oil production in developing seeds of *Prunus sibirica* for woody biodiesel

**DOI:** 10.1186/s13068-018-1347-x

**Published:** 2019-01-04

**Authors:** Jia Wang, Weijun Lin, Zhongdong Yin, Libing Wang, ShuBin Dong, Jiyong An, Zixin Lin, Haiyan Yu, Lingling Shi, Shanzhi Lin, Shaoliang Chen

**Affiliations:** 1 Beijing Advanced Innovation Center for Tree Breeding by Molecular Design, College of Biological Sciences and Biotechnology, School of Soil and Water Conservation, National Engineering Laboratory for Tree Breeding, Beijing Forestry University, Beijing, 100083 China; 2 Institute of Botany, Chinese Academy of Sciences, Beijing, 100093 China; 3 Research Institute of Forestry, Chinese Academy of Forestry, Beijing, 100091 China

**Keywords:** *Prunus sibirica* seeds, Biodiesel fuel properties, Oil synthesis, Intracellular transporter, Differential transcript profiles, Transporter-mediated mechanism

## Abstract

**Background:**

Based on our previous studies of 17 *Prunus sibirica* germplasms, one plus tree with high quality and quantity of seed oils has emerged as novel potential source of biodiesel. To better develop *P. sibirica* seed oils as woody biodiesel, a concurrent exploration of oil content, FA composition, biodiesel yield and fuel properties as well as prediction model construction for fuel properties was conducted on developing seeds to determine the optimal seed harvest time for producing high-quality biodiesel. Oil synthesis required supply of carbon source, energy and FA, but their transport mechanisms still remains enigmatic. Our recent 454 sequencing of *P. sibirica* could provide long-read sequences to identify membrane transporters for a better understanding of regulatory mechanism for high oil production in developing seeds.

**Results:**

To better develop the seed oils of *P. sibirica* as woody biodiesel, we firstly focused on a temporal and comparative evaluation of growth tendency, oil content, FA composition, biodiesel yield and fuel properties as well as model construction for biodiesel property prediction in different developing seeds from *P. sibirica* plus tree (accession AS-80), revealing that the oils from developing seeds harvested after 60 days after flowering (DAF) could be as novel potential feedstock for producing biodiesel with ideal fuel property. To gain new insight into membrane transport mechanism for high oil yield in developing seeds of *P. sibirica*, we presented a global analysis of transporter based on our recent 454 sequencing data of *P. sibirica*. We annotated a total of 116 genes for membrane-localized transporters at different organelles (plastid, endoplasmatic reticulum, tonoplast, mitochondria and peroxisome), of which some specific transporters were identified to be involved in carbon allocation, metabolite transport and energy supply for oil synthesis by both RT-PCR and qRT-PCR. Importantly, the transporter-mediated model was well established for high oil synthesis in developing *P. sibirica* seeds. Our findings could help to reveal molecular mechanism of increased oil production and may also present strategies for engineering oil accumulation in oilseed plants.

**Conclusions:**

This study presents a temporal and comparative evaluation of developing *P. sibirica* seed oils as a potential feedstock for producing high-quality biodiesel and a global identification for membrane transporters was to gain better insights into regulatory mechanism of high oil production in developing seeds of *P. sibirica*. Our findings may present strategies for developing woody biodiesel resources and engineering oil accumulation.

**Electronic supplementary material:**

The online version of this article (10.1186/s13068-018-1347-x) contains supplementary material, which is available to authorized users.

## Background

Biodiesel, one of the renewable energy sources, has characterized as ecofriendly fuel with biodegradability and nontoxicity [1]. In recent years, green diesel produced from seed oils of several woody plants has been shown with a notable advantage over conventional feedstock in China [2–8].

Siberian apricot (*Prunus sibirica* L.), small deciduous tree of the family Rosaceae and the genus *Prunus*, is widely distributed in China with the total area of about 1,700,000 ha, and the annual seed production is above 192,500 metric tons [9–11]. There are many germplasms of *P. sibirica* with different oil yield and quality in China, of which some accessions have been identified with rich oil content and high proportion of linoleic and oleic acid [12], and the mean oil content (50.5%) of mature dry seeds from 17 different germplasms was higher than that of Chinese traditional woody oil plants (such as *Vernicia Montana*, *Sapium sebiferum*, *Pistacia chinensis*, *Lindera glauca, Jatropha curcas* and *Comus wilsoniana*) [3, 7, 8, 13–15]. All these revealed that *P. sibirica* seed oils may be as a novel potential source of biodiesel feedstock in China. To provide a schedule for optimal harvest time of developing seeds with high-quality oils for better development of woody biodiesel, it is important to evaluate dynamic changes of oil content, FA composition, biodiesel yield and fuel property in developing *P. sibirica* seeds.

In oilseed plants, de novo FA synthesis and its elongation as well as triacylglycerol (TAG) assembly are known to occur in different subcellular organelles, but the end products of both plastidial FA synthesis and cytosolic FA elongation are transferred to the endoplasmic reticulum (ER) for TAG assembly [16, 17]. In general, sucrose as main source of carbon available for oil synthesis is stored in vacuole [17–19], which is needed to be transported into sink organs (such as fruit and seed), and then converted to key precursor (pyruvate) for FA synthesis in both cytosol and plastid [16, 20, 21]. In addition, de novo FA synthesis in plastid required energy (reducing power and ATP) from several metabolic pathways in different organelles [22]. All these have revealed a complex network of carbon partitioning and energy provision for plant oil synthesis at subcellular level, thus requiring several specific membrane transporters [23–25]. In recent years, plastidial transporters have been ascribed a key role in carbon allocation and adenine nucleotide transport [26–32], and mitochondrial transporters implicated in ATP synthesis has been identified in plants [24, 33–35]. Also, several transport proteins have been shown to transport of lipid, FA or acyl-CoA [36–48], such as ATP-binding cassette (ABC) protein, acyl-CoA-binding protein (ACBP) and lipid transfer protein (LTP). Yet, the transporter-mediated mechanism of carbon source allocation and energy supply available for FA synthesis and oil accumulation still remains enigmatic. To develop the *P. sibirica* seed oils as woody biodiesel, our previous work focused on overall analyses of Illumina-sequencing data and lipid gene expression in developing seeds of *P. sibirica*, but we found that some short sequences were not so effective to get BLAST hits owing to lack of a characterized protein domain [11]. Recently, we have performed 454 deep sequencing analysis of *P. sibirica* (SRX339392) [49], from which the provision of numerous longer database may make us possible to globally annotate transporters specific for oil synthesis in developing seeds of *P. sibirica*.

The aim of this sequential study was to better develop seed oils of *P. sibirica* as woody biodiesel. To this end, we selected one ideal germplasm (accession AS-80) with high quality and quantity of seed oils as experimental materials, and then focused on a temporal evaluation of growth tendency (size and weight), oil accumulation (content and composition), biodiesel yield and fuel properties of developing seeds from 10 DAF (immature stage) to 70 DAF (fully mature stage). Also, a triangular model was established for biodiesel property prediction of raw seed oils during development. Such assessment could provide vital information for selecting the optimal harvest time of developing seeds to obtain high-quality biodiesel. On the other hand, we performed a global analysis of membrane transporters as an attempt to gain new insights into molecular mechanism of high seed oil synthesis for development of woody biodiesel. Based on our previous 454 sequencing data of *P. sibirica*, we annotated the genes encoding for membrane transporters in various organelles (peroxisome, mitochondria, plastid, vacuole and ER). Finally, by both qRT-PCR and RT-PCR detections, some key transporters were identified to be involved in carbon source allocation and energy provision as well as metabolite transport for FA synthesis, TAG assembly and mobilization in developing seeds, with the aim of deriving a transporter-mediated metabolic model for high oil production. Our findings could provide a better understanding of oil accumulative molecular mechanism and also present new biotechnological targets to improve seed oil yields for the development of woody biodiesel.

## Result

### Temporal analysis of growth tendency for developing seeds

**Fig. 1:**
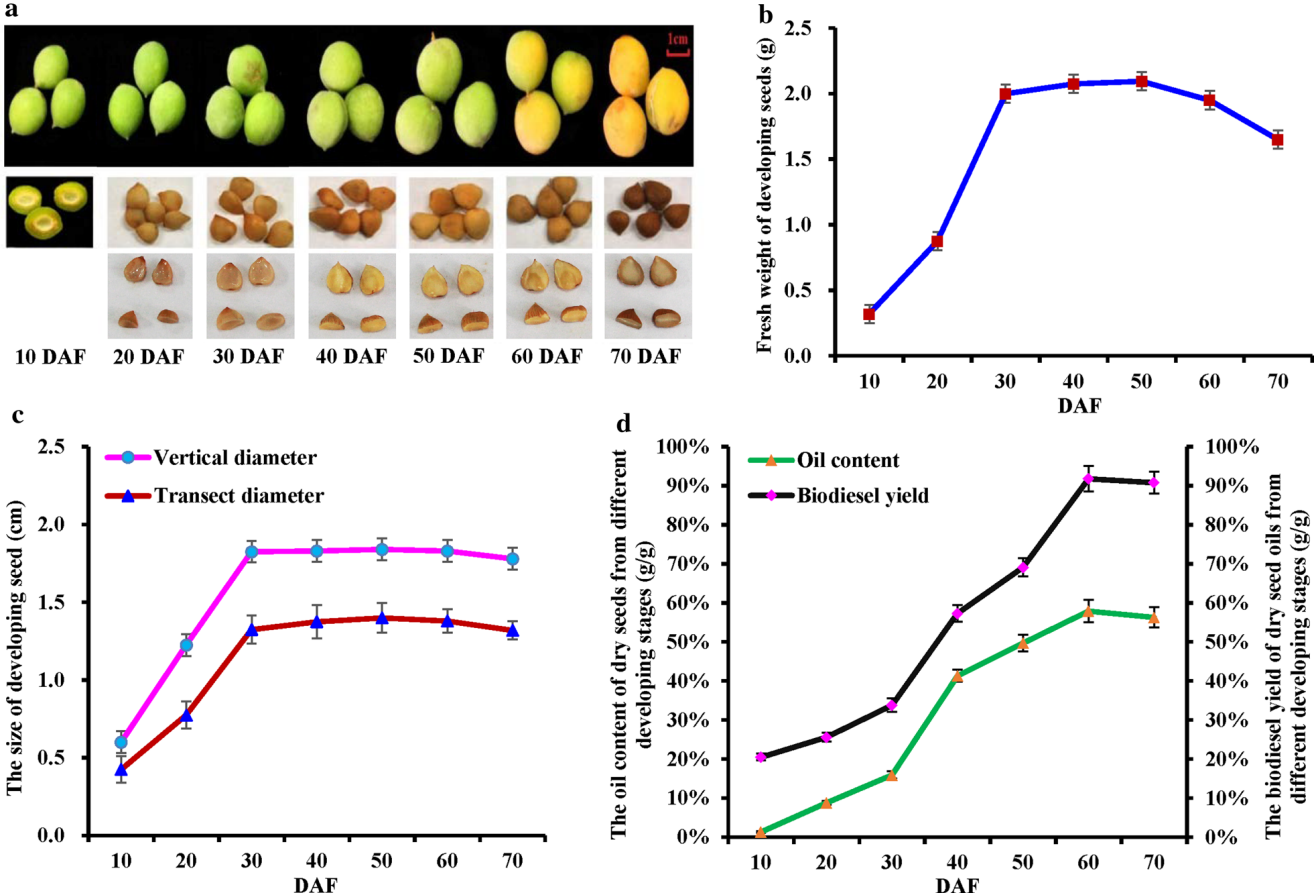
Dynamic changes of oil accumulation and biodiesel yield in developing *P. sibirica* seeds. **a** The feature of *P. sibirica* seeds from seven developing stages. **b** Dynamic change of the seed fresh weight during development. **c** Dynamic change of seed size (longitudinal and transverse diameter) during development. **d** Dynamic changes in oil content and biodiesel yield of dry seeds from different developing stages. Error bars are standard deviations (SD) of three biological replicates

To develop the seed oils of *P. sibirica* as woody material for biodiesel production in China, we selected one plus tree (germplasm accession AS-80) with rich seed oils as specific experimental material. Given the fruit growth of *P. sibirica* in response to different developing stages (Fig. 1a), we analyzed dynamic changes of growth tendency (size and fresh weight) for developing seeds from 10 DAF (immature stage) to 70 DAF (full mature stage). It was observed that the fresh weight of developing seeds was about 5.3-fold higher at 30 DAF than at 10 DAF, and 4.8% increase was detected at 40–50 DAF, followed by 13.1% decline at 60–70 DAF (Fig. 1b), as was the case for seed size (transverse diameter and longitudinal) during development (Fig. 1c), indicating that developing seeds of *P. sibirica* had almost attained their final sizes at early middle stage (10–50 DAF).

### Dynamic changes of oil accumulation and biodiesel yield of developing seeds

The above results prompted us to explore the relationship between oil accumulation and growth of developing seeds. We detected the dynamic patterns of oil content and FA compositions as well as biodiesel yield during seed development and found a gradual increase in oil content of dry seeds at 10–60 DAF (1.36–57.93%) with a rapid accumulation during 40–60 DAF, but 2.1% decline was detected at 70 DAF (full rape) (Fig. 1d), revealing an active oil synthesis of developing seeds at the middle-late stage (40–60 DAF). Given a rapid phase of seed growth at early middle stage (10–30 DAF) (Fig. 1a–c), it seems clear that a main growth of seeds started before active oil accumulation during development. In addition, we identified 9 kinds of FA compositions in developing seed oils (Table 1), and both oleic acid (C18:1) and linoleic acid (C18:2) were the predominant compounds, of which the C18:1 content increased from 10 DAF (11.59%) to 70 DAF (76.39%), but an opposite trend was noted for C18:2 (from 57.95 to 18.49%). The linolenic acid (C18:3) showed peak value (10.51%) at 40 DAF, and palmitic acid (C16:0) exhibited a decrease from 19.92 to 2.86% during seed development. However, myristic acid (C14:0) was detected only at 10 DAF, and the other saturated or long-chain FAs with lower proportion showed no notable change during seed development. These data indicated an obvious variation of oil contents and FA compositions in developing seeds (Table 1; Fig. 1d), which allowed us to explore the feature of biodiesel yield during seed development. It was found that the biodiesel yield of developing seed oils was about 3.5-fold higher at 60 DAF (91.8%) than at 10 DAF (20.5%), while 1.2% decline was detected at 70 DAF (Fig. 1d). Of note, the average yield of biodiesel (91.3%) at 60–70 DAF was higher than that of other *P. sibirica* germplasms (89.7%) [3, 8], implying that the seeds oils from late developing *P. sibirica* may be suitable for producing high yield biodiesel.

**Table 1:** Dynamic changes of FA compositions and their relative proportions in developing seeds of *P. sibirica*

DAF	C14:0 (%)	C16:0 (%)	C16:1 (%)	C18:0 (%)	C18:1 (%)	C18:2 (%)	C18:3 (%)	C20:0 (%)	C20:1 (%)
10	0.71 ± 0.01	19.92 ± 1.58	0.94 ± 0.09	2.51 ± 0.51	11.59 ± 0.91	57.95 ± 1.21	6.38 ± 0.42	–	–
20	–	11.30 ± 1.07	1.05 ± 0.11	3.01 ± 0.81	21.80 ± 1.05	52.79 ± 1.01	9.80 ± 0.63	0.11 ± 0.02	0.14 ± 0.05
30	–	5.02 ± 0.82	0.81 ± 0.04	4.97 ± 0.51	27.14 ± 1.02	51.62 ± 1.22	10.23 ± 0.71	0.10 ± 0.03	0.11 ± 0.01
40	–	4.11 ± 0.51	0.70 ± 0.05	1.50 ± 0.07	32.51 ± 1.03	50.49 ± 1.01	10.51 ± 0.61	0.08 ± 0.01	0.10 ± 0.02
50	–	3.92 ± 0.23	0.61 ± 0.01	1.24 ± 0.02	36.42 ± 1.81	47.14 ± 1.02	10.43 ± 0.54	0.13 ± 0.02	0.11 ± 0.01
60	–	2.91 ± 0.32	0.61 ± 0.02	1.07 ± 0.03	72.22 ± 2.01	22.47 ± 0.98	0.51 ± 0.03	0.09 ± 0.04	0.12 ± 0.04
70	–	2.86 ± 0.13	0.59 ± 0.01	1.01 ± 0.01	76.39 ± 3.02	18.49 ± 0.71	0.39 ± 0.01	0.14 ± 0.03	0.13 ± 0.02

Error bars are standard deviations (SD) of three biological replicates

### Construction of prediction model for biodiesel fuel properties of raw oils from developing seeds

**Fig. 2:**
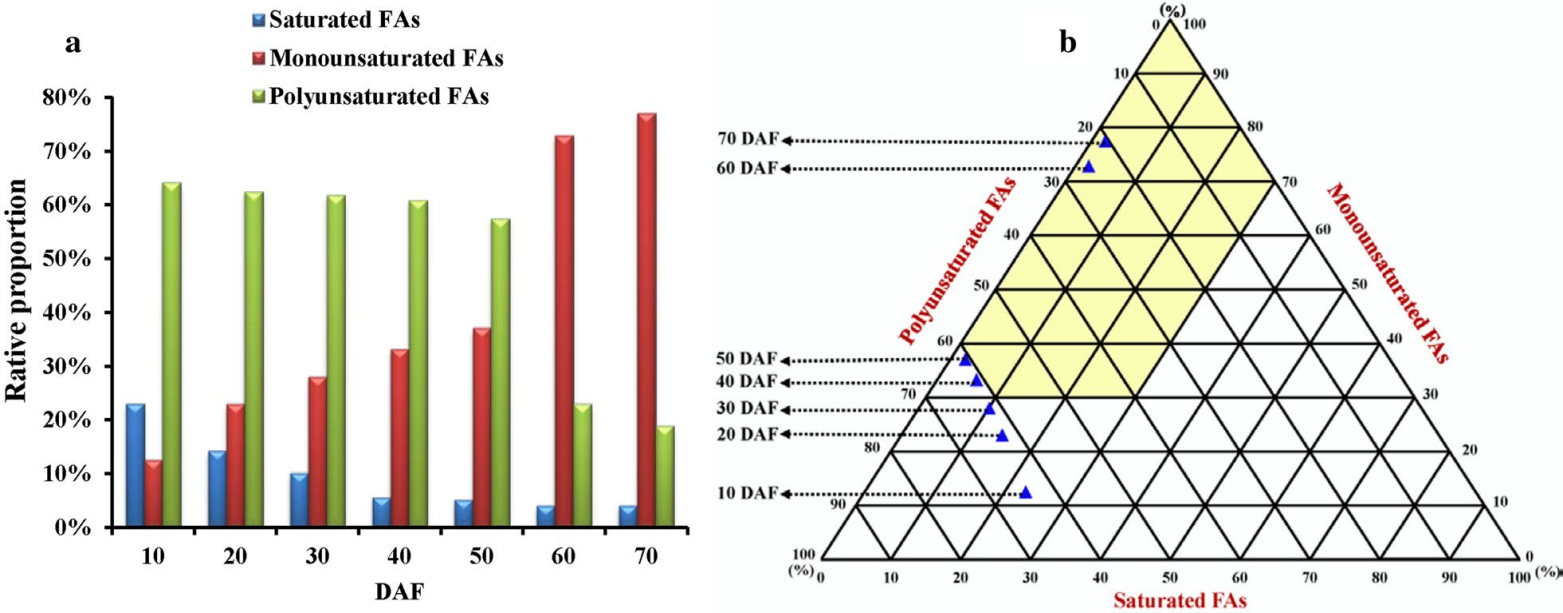
Construction of prediction model for biodiesel fuel properties of raw oils from developing *P. sibirica* seeds. **a** Change of relative proportion of saturated, monounsaturated and polyunsaturated FAs in developing *P. sibirica* seeds. **b** Prediction triangular chart of FA compositions on biodiesel fuel properties. The yellow part of region was clearly delineated to predict the biodiesel fuel properties that could fully meet the limit of cetane number, iodine number, cold filter plugging point and oxidation stability. Error bars are standard deviations (SD) of three biological replicates

Given a serious phenomenon of harvesting the not-fully matured *P. sibirica* seeds in China [50], together with our finding of notable variation for oil content (1.36–57.93%) and total proportion of C18:1 and C18:2 (69.54–94.88%) during seed development (Table 1), it was essential for us to predict biodiesel fuel properties of developing seed oils for determining the optimal harvest time. Most properties of biodiesel fuel are known to be dependent on the compositions and amounts of FAs in raw oils [51–53]. Recently, based on the influence of FA compositions on fuel properties of mature dry seed oils from 10 woody plants, we have constructed a triangular predict model for fuel properties of biodiesel from raw oils [50]. For this study, the percentages of polyunsaturated, monounsaturated and saturated FAs in different developing seed oils were calculated (Fig. 2a) and then a triangular predict graph was drawn (Fig. 2b), in which the yellow region that could satisfy the limit of cetane number (CN), iodine number (IN), oxidation stability (OS) and cold filter plugging point (CFPP) [50] was delineated to predict the biodiesel fuel properties. Our finding that developing seeds at 60–70 DAF were allocated into yellow area of triangular graph (Fig. 2b) revealed that the oils from late developing seeds (60–70 DAF), as raw material for biodiesel, could meet the fuel properties. Considering the highest oil content (57.82%) and the near-maximal total percentage of C18:1 and C18:2 (94.71%) as well as good yield of biodiesel (91.6%) detected in developing seeds at 60 DAF (Table 1; Fig. 1d), we recommended that the optimal time for harvesting *P. sibirica* seeds was at 60 DAF to produce high yield biodiesel with superior fuel properties.

### Temporal evaluation of biodiesel fuel properties of oils form developing seeds

To better determine a schedule for harvesting the developing *P. sibirica* seeds with high quality of biodiesel, we also analyzed the dynamic changes of biodiesel fuel properties of FA methyl esters (FAMEs) from seed oils during development, including IV, CN, CFPP, OS, cloud point (CP), density (*D*) and kinematic viscosity (KV). Our detected values of IV (102.76–119.75), *D* (876.09–882.78) and KV (4.08–4.48) of FAMEs were within the ranges specified by USA (ASTM D6751-2010: IN < 120, 1.9 < KV < 6.0, no stipulate density limit), European (EN 14214-2008: IV < 120, 860 < *D*<900, 3.5 < KV < 5.0) and China (GB/T20828-2007: IV < 120, 820 < *D*<900, 1.9 < KV < 6.0) (Table 2). However, the values of OS (3.3 h) and CN (51.6) of biodiesel only from late developing seeds (60–70 DAF) could satisfy the standards of ASTM D6751-2010 (OS > 3.0 h) and EN 14214-2008 (51 < CN < 65), respectively. Both CP and CFPP are two important low-temperature parameters, but not limited by the EN 14214 standard. In this work, the CP value (− 3.99 to − 9.81 °C) of developing seeds was lower than ASTM D6751-2010 (− 3 °C < CP < 12 °C), and the CFPP valve (− 2.18 ∼ − 12.48 °C) (Table 2) was less than the maximum limit (0 °C) of Germany standard (DIN V51606-1997) in summer, of which − 12.5 °C from late developing seeds (60–70 DAF) was lower than the minimum limit (− 10.0 °C) for both spring and autumn, implying a better cold flow property for biodiesel production from seed oils of late developing stage. In addition, a lower content (0.39–10.51%) of C18:3 and the FAMEs with no four double bonds (Table 1) all satisfied the EN 14214-2008 specification (< 12% and 1%, respectively). These results, together with temporal evaluations of oil content, FA composition, biodiesel yield and fuel property (Fig. 1d; Table 1), revealed that the *P. sibirica* seeds harvested after 60 DAF was well suitable for producing biodiesel also corresponded to our constructed triangular prediction model for biodiesel fuel properties of developing seeds (Fig. 2b).

**Table 2:** Evaluation of biodiesel fuel properties of oils from different developing seeds of *P. sibirica*

DAF	Biodiesel fuel properties								
	DU	LCSF	CN	IV (g/100 g)	CFPP (°C)	CP (°C)	OS (h)	KV (mm^2^ s^−1^, 40 °C)	D (kg/m^−3^, 15 °C)
10	147.72 ± 3.1	7.17 ± 0.8	47.24 ± 3.5	123.76 ± 2.5	− 2.18 ± 0.1	0.32 ± 0.1	2.32 ± 0.02	4.08 ± 0.09	882.75 ± 1.21
20	158.19 ± 2.6	4.67 ± 0.7	45.97 ± 3.8	130.75 ± 2.8	− 6.57 ± 0.1	− 3.99 ± 0.1	2.76 ± 0.03	4.14 ± 0.10	881.18 ± 1.05
30	162.13 ± 2.4	3.53 ± 0.2	45.49 ± 1.2	133.40 ± 1.5	− 8.58 ± 0.2	− 5.97 ± 0.2	2.90 ± 0.01	4.23 ± 0.11	879.39 ± 1.31
40	162.80 ± 2.2	1.89 ± 0.1	45.81 ± 0.9	133.38 ± 1.3	− 11.46 ± 0.3	− 8.79 ± 0.1	2.91 ± 0.01	4.28 ± 0.08	876.28 ± 0.89
50	165.29 ± 2.1	1.75 ± 0.3	45.11 ± 0.8	135.49 ± 1.1	− 11.71 ± 0.1	− 9.03 ± 0.2	2.94 ± 0.04	4.32 ± 0.11	878.45 ± 0.91
60	116.31 ± 1.5	1.30 ± 0.2	51.58 ± 0.7	105.65 ± 0.5	− 12.47 ± 0.1	− 9.81 ± 0.1	3.30 ± 0.01	4.36 ± 0.08	877.57 ± 0.97
70	115.63 ± 1.5	1.31 ± 0.3	51.71 ± 0.6	102.35 ± 1.5	− 12.48 ± 0.2	− 9.79 ± 0.1	3.33 ± 0.01	4.48 ± 0.10	876.09 ± 1.01

*DU* degree of unsaturation, *LCSF* chain length saturated factor, *CN* cetane number, *IV* iodine value, *CFPP* cold filter plugging point, *CP* cloud point, *OS* oxidation stability, *KV* kinematic viscosity, *D* density. Error bars are standard deviations (SD) of three biological replicates

### Genome-wide identification of genes for the transporters in developing seeds

In oilseed plants, de novo FA synthesis and its elongation as well as TAG assembly in multiple organelles required supply of energy, carbon source, or end product across membranes by selective transporters [16, 24, 36, 48], but the transport mechanism remains one of most interesting open challenges encountered in study of oil synthesis. Recently, the provision of numerous longer reads from our 454 deep sequencing of *P. sibirica* [49] may make us possible to globally annotate the genes for transporters by BLASTX against the public databases (Additional file 1: Table S1). A total of 116 genes were identified for membrane transporters in various organelles (plastid, vacuole, peroxisome, mitochondria and ER), mostly implicated in FA synthesis, TAG assembly and mobilization, including the interchange of glycolytic intermediate, transport of sucrose, acyl-CoA, adenine nucleotide and FA, and energy supply (ATP synthesis and TCA cycle) as well as oil mobilization (TAG hydrolysis, FA β-oxidation, glyoxylate cycle and gluconeogenesis) (Additional files 2, 3, 4, 5, 6, 7: Table S2, S3, S4, S5, S6, S7), implying a complex transporter-mediated mechanism of carbon allocation and energy supply for oil synthesis in developing seeds of *P. sibirica*. In the following, our work focused on the analysis of temporal transcript profiles for all annotated transporters by both qRT-PCR and RT-PCR during seed development to identify transporters specific for high oil production.

### Temporal transcripts of plastidial transporters for carbon allocation and ATP transport in developing seeds

**Fig. 3:**
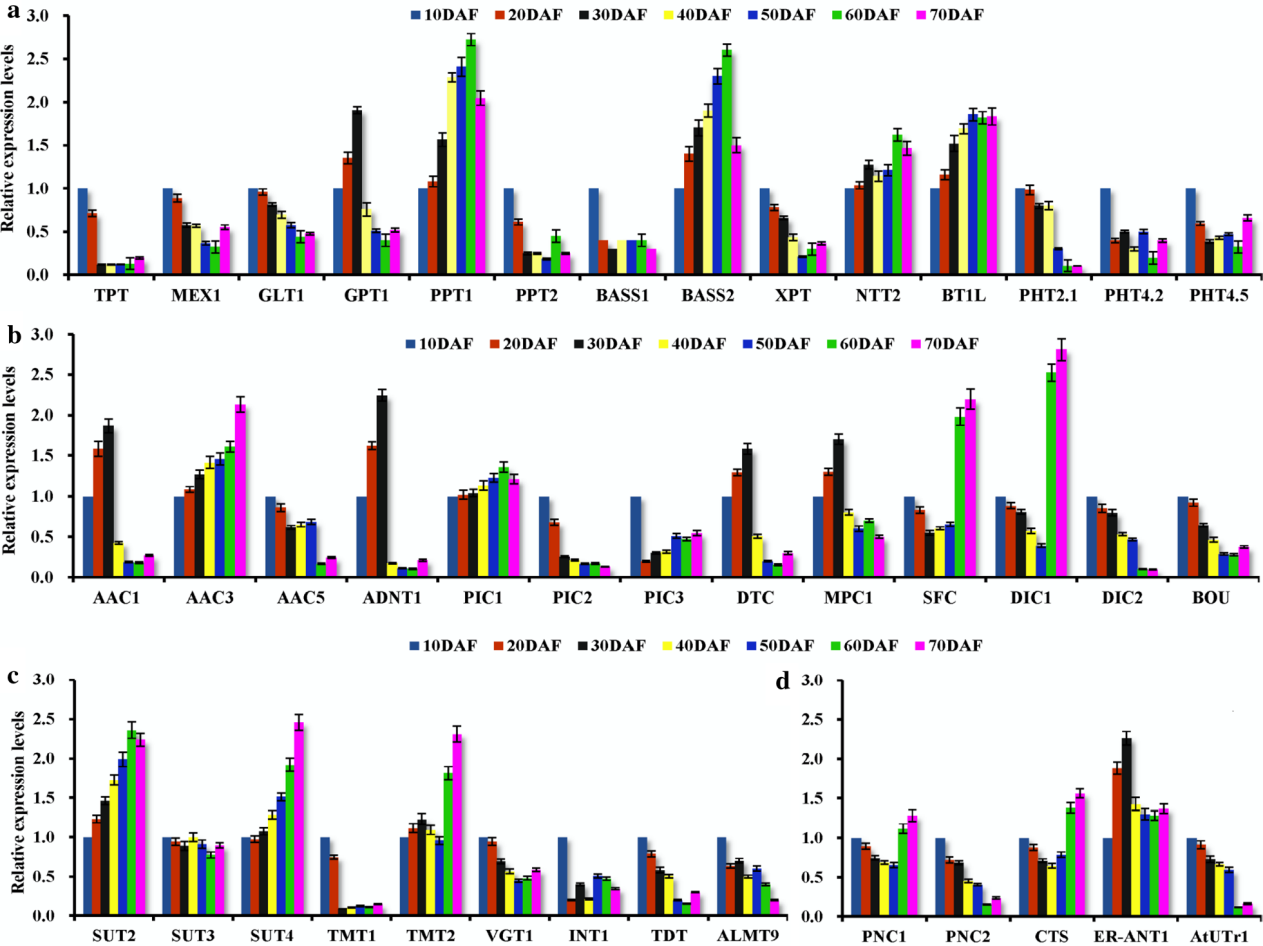
Transcriptional analysis of transporters for carbon allocation and energy provision in developing *P. sibirica* seeds by qRT-PCR. **a** Temporal transcript profiles for transporters of plastid membrane involved in carbon allocation and metabolite transport. **b** Temporal transcript profiles for mitochondrial transporters implicated in TCA cycle, ATP synthesis and oil mobilization. **c** Temporal transcript profiles for transporters of tonoplast involved in sugar transport. **d** Temporal transcript profiles for transporters of peroxisomal and ER membrane involved in oil synthesis and mobilization. The genes for cyclophilin (CYP) and ubiquitin-conjugating enzyme (UBC) were used as internal controls. Expression level from seed sample at 10 DAF was arbitrarily set to 1.00 for standardization. Error bars are SD of three biological replicates with three technical repetitions each

**Fig. 4:**
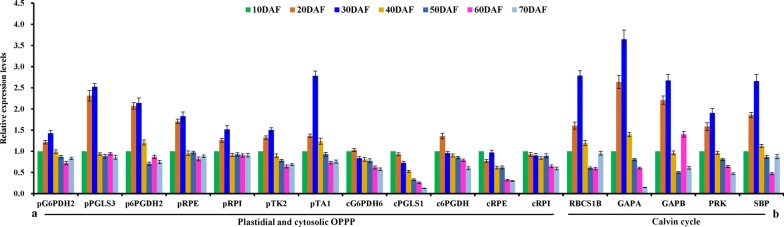
Transcriptional analysis of enzymes for carbon assimilation and OPPP in developing *P. sibirica* seeds by qRT-PCR. **a** Comparative analysis of transcript levels for enzymes involved in both cytosolic and plastidial OPPP. **b** Differential transcript for genes involved in carbon assimilation of Calvin cycle. Both *CYP* and *UBC* genes were used as internal controls. Expression level from seed sample at 10 DAF was arbitrarily set to 1.00 for standardization. The cytosolic (c) and plastidial (p) isoforms of the enzymes are indicated by a prefix in **a**. Error bars are SD of three biological replicates with three technical repetitions each

Plastid transporters have been shown for interchange of glycolytic intermediates between cytosol and plastid in plants [30–32], including glycolipid transporter (GLT), xylulose 5-phosphate transporter (XPT), maltose exporter (MEX), glucose-6-phosphate (G6P) transporter (GPT), phosphoenolpyruvate transporter (PPT), triose phosphate transporter (TPT), and bile acid/sodium symporter (BASS, PYR carrier), of which the orthologs of BASS1/2, MEX1, PPT1/2, XPT, GLT1, TPT, PPT and GPT1 were identified (Additional file 2: Table S2). Our detections of qRT-PCR and RT-PCR indicated that GPT1 exhibited high transcript at 10–30 DAF, and PPT1/BASS2 transcript increased during seed development, but less transcript was marked for others (Additional file 8: Figure S1a; Fig. 3a), implying that BASS2/GPT1/PPT1 may contribute to allocate cytosolic glycolytic metabolite (G6P, PEP or PYR) into plastid during seed development. In addition, the finding of rich transcript for all enzymes of plastid OPPP at 10–30 DAF (Fig. 4a) assumed a role of plastid OPPP for FA synthesis in early developing seeds. Also note was plastid homologies for PHT2.1/4.2/4.5 (Pi transporter), BT1L (adenine nucleotide uniporter) and NTT2 (ATP/ADP antiporter) (Additional file 2: Table S2), but only both NTT2 and BT1L showed transcript abundance (Fig. 3a; Additional file 8: Figure S1a), pointing to a role of NTT2/BT1L in transport adenine nucleotide during seed development.

### Transcript analysis of mitochondrial transporters for ATP synthesis and oil metabolism in developing seeds

By functional annotation, some orthologs of mitochondrial transporters were marked (Additional file 3: Table S3), including acetyl-carnitine/carnitine carrier (BOU), dicarboxylate/tricarboxylate carrier (DTC), adenine nucleotide transporter (ADNT1), ADP/ATP carrier (AAC1/3/5), phosphate carrier (PIC1/2/3), dicarboxylate carrier (DIC1/2), PYR carrier (MPC1) and succinate/fumarate carrier (SFC). Analyses of differential profiles showed that ACC3/PIC1 transcript was up-regulated during seed development, and ADNT1/DTC/ACC1/MPC1 transcript enhanced before 30 DAF, while ACC5 and PIC2/3 displayed low transcript (Fig. 3b; Additional file 8: Figure S1c), indicating that DTC MPC1, AAC1/3, ADNT1 and PIC1 may be as main transporters for PYR transport or ATP synthesis in developing seeds. In addition, the transporters for oil mobilization were detected with differential transcripts, of which SFC and DIC1 exhibited high transcript at 60–70 DAF, but a strong decline of transcript was observed for BOU (Fig. 3b), suggesting a specific contribution of DIC1/SFC to oil mobilization in late developing seeds.

### Temporal transcripts for tonoplast transporters involved in sugar transport in developing seeds

Given vacuole as temporary reservoir for many metabolites [19, 54], we attempted to explore the possibility of tonoplast transporter in delivery them as carbon source for FA synthesis in developing seeds. Here, the genes for orthologs of glucose transporter (TMT1/2 and VGT1, cytosol glucose import), inositol transporter (INT1), malate transporter (ALMT9), dicarboxylate transporter (TDT) and sucrose symporter (SUT2/3/4, vacuole sucrose export) were annotated (Additional file 4: Table S4), implying a diversity of tonoplast metabolite transport in developing seeds. However, only SUT2/4 showed high transcript during development, and TMT2 increased transcript after 60 DAF (Additional file 8: Figure S1d; Fig. 3c), suggesting that SUT2/4 may be specific for export vacuole sucrose into cytosol during seed development, but TMT2-mediated glucose uptake into vacuole was at maturing stage.

### Transcript analysis of ATP and nucleotide sugar transporters of ER membrane in developing seeds

It was shown that ER membrane-localized ATP transporter (ER-ANT1) and UDP-glucose transporter (AtUTr) may play a role for seed development and oil increase of Arabidopsis [55, 56]. Here, a combination of annotation and transcript detection indicated that ER-ANT1 transcript increased before 30 DAF and then maintained abundance toward mature stage (Additional file 5: Table S5; Additional file 8: Figure S1b; Fig. 3d), which was correlated with seed growth tendency during development (Fig. 1b, c), refeclting that ANT1-mediated ATP import into ER may be essential for seed growth. However, the finding of reduced AtUTr1 transcript (Fig. 3d) suggested its unimportance for the UDP-glucose transport during seed development.

### Transcript analysis of transporters of peroxisomal membrane for TAG mobilization in developing seeds

**Fig. 5:**
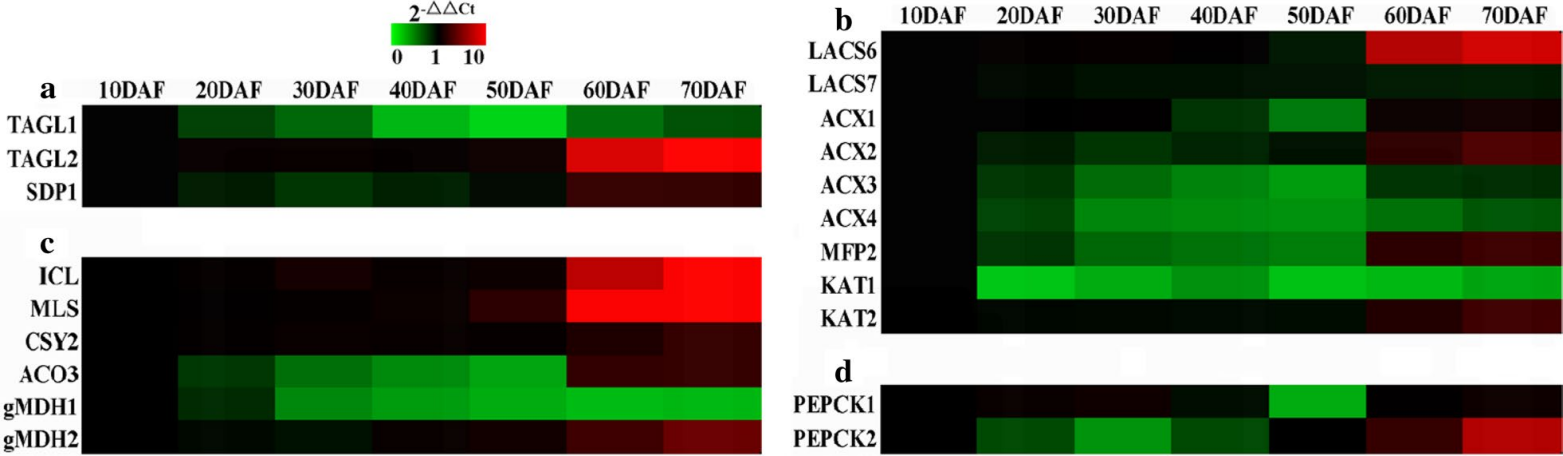
Transcriptional analysis of enzymes for TAG mobilization in developing *P. sibirica* seeds by qRT-PCR. **a** Temporal transcript patterns of TAG lipases (TAGL1/2) and sugar dependent 1 (SDP1) protein involved in TAG hydrolysis. **b** Temporal transcript patterns of enzymes involved in β-oxidation. **c** Temporal transcript patterns of enzymes for glyoxylate cycle. **d** Temporal transcript patterns of PEP carboxykinase (PEPCK1/2) involved in gluconeogenesis. Both *CYP* and *UBC* genes were used as the internal controls. The relative expression values in heatmap were counted as 2−^△△Ct^, and expression level from seed sample at 10 DAF was arbitrarily set to 1.00 for standardization

Given 2.1% decline detected for seed oil content at 70 DAF (Fig. 1d), we explored the nature of TAG mobilization during development. By annotation and profile analysis, the genes for oil body-related sugar dependent 1 (SDP1) and TAG lipase (TAGL2), and peroxisome-localized PNC1/2 (adenine nucleotide carrier) and CTS (FA transporter) were identified (Additional file 7: Table S7), most of which were up-regulated before 70 DAF (Additional file 8: Figure S1b; Figs. 3d, 5a), revealing an initiation of TAG hydrolysis and main role of PNC/CTS in import of ATP and FA into peroxisome in late developing seeds, as allowed us to evaluate FA β-oxidation during seed development. The orthologs for enzymes of β-oxidation [multifunctional protein (MFP2), acyl-CoA oxidase (ACX1/2), long-chain acyl-CoA synthetase (LACS6/7) and 3-ketoacyl-CoA thiolase (KAT1/2)], glyoxylate cycle [aconitase (ACO3), citrate synthase (CYS2), Mal synthase (MLS), dehydrogenases (MDH1/2) and isocitrate lyase (ICL)], and gluconeogenesis [PEP carboxykinase (PEPCK1/2)] were marked with differential transcripts, of which LACS6, MFP2, KAT2, PEPCK2, ACX1/2, MDH2, CYS2, ACO3, ICL and MLS showed rich transcription at 60–70 DAF (Additional file 7: Table S7; Fig. 5b–d), implying that β-oxidation, gluconeogenesis and glyoxylate cycle were active only in late developing seeds.

### Transcript analysis of transport proteins involved in FA transport for TAG synthesis in developing seeds

**Fig. 6:**
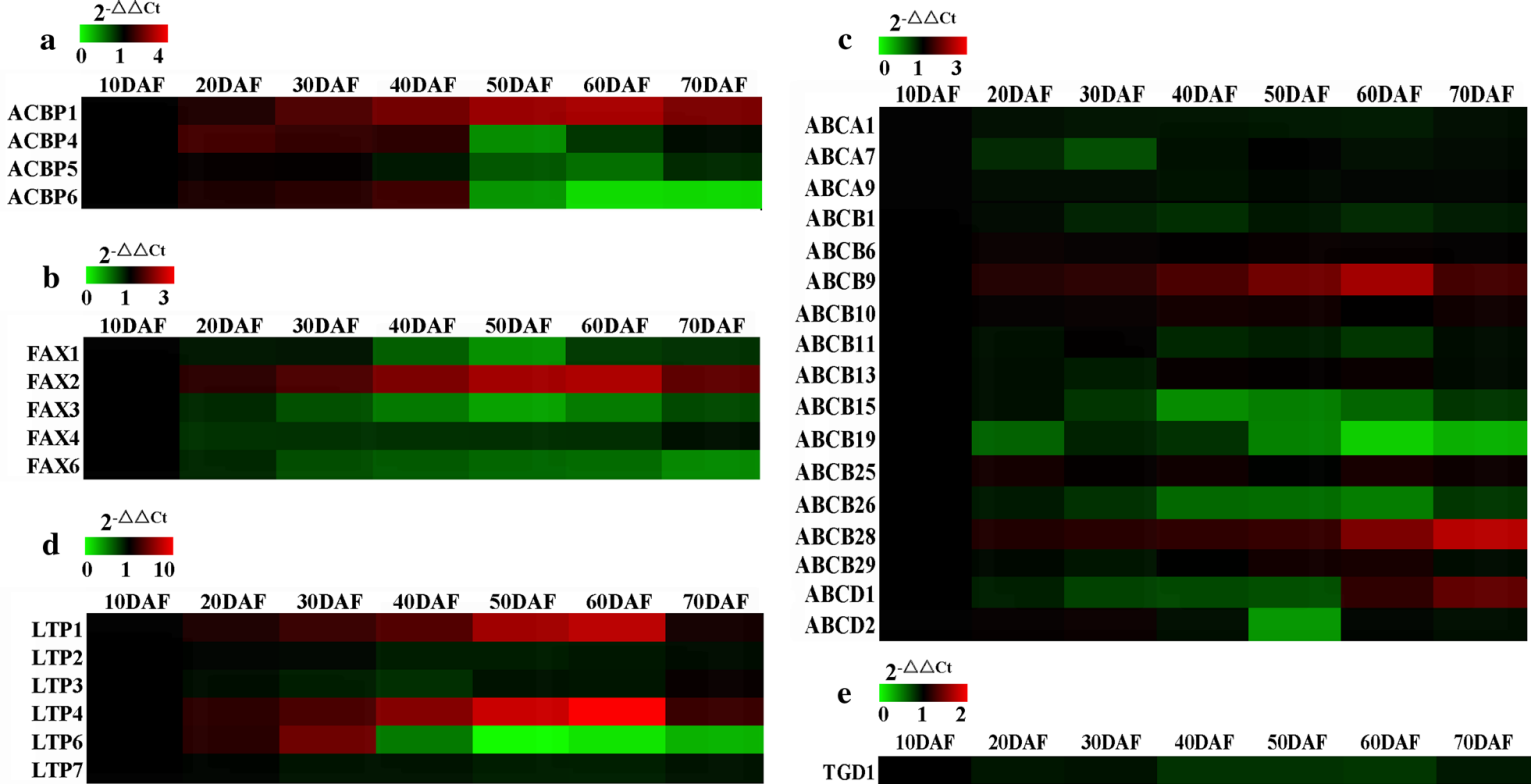
Transcriptional analysis of transport proteins for TAG synthesis in developing *P. sibirica* seeds by qRT-PCR. **a** Temporal transcript patterns of genes encoding for acyl-CoA-binding proteins (ACBPs). **b** Temporal transcript patterns of genes for membrane protein of fatty acid export (FAX). **c** Temporal transcript patterns of genes for some important ATP-binding cassette (ABC) proteins. **d** Temporal transcript patterns of genes for lipid transfer proteins (LTPs). **e** Temporal transcript pattern of gene for trigalactosyldiacylglycerol 1 (TGD1) protein. Both *CYP* and *UBC* genes were used as the internal controls. The relative expression values in heatmap were counted as 2−^△△Ct^, and the expression level from seed sample at 10 DAF was arbitrarily set to 1.00 for standardization

It was reported that acyl-CoA-binding protein (ACBP) and FA export (FAX) protein may contribute to FA import into ER for TAG synthesis in oilseed plants [39, 40, 48, 57, 58]. The genes for cytosolic ACBP4/5/6, and plastidial FAXs (FAX1-4, 6) and ACBP1 were noted with differential transcripts, of which ACBP1 and FAX2 showed abundant transcript during seed development, but ACBP4/6 transcript was higher only at 10–40 DAF (Fig. 6a; Additional file 8: Figure S1e; Additional file 6: Table S6), inferring that ACBP1/4/6 and FAX2 may be potential candidates for FA transport during TAG synthesis of developing seeds. We also annotated 63 genes for ABC transporter family, of which 3, 12, 12, 2, 1, 4, 23 and 6 members were, respectively, assigned to 8 known subfamilies (ABCA, B, C, D, E, F G and I) (Additional file 6: Table S6), revealing a diversity of ABC transporters in developing seeds of *P. sibirica*. Given a role of ABCA/B/D subfamily in transport FA or lipid [37], we analyzed temporal transcripts for members of ABCA/B/D subfamily in developing seeds, but only both ABCD1 and ABCB9/28 displayed rich transcription (Fig. 6c; Additional file 8: Figure S1e). Another was concerned about trigalactosyldiacylglycerol (TGD) protein and lipid transfer protein (LTP) in regulation seed growth [41, 59]. Of our identified TGD1 and 6 LTPs (LTP1/2/3/4/6/7) (Additional file 6: Table S6), LTP6 and LTP1/4 transcripts were up-regulated before 30 and 60 DAF, respectively, whereas down-regulated transcript was detected for TGD1 and LTP2/3/7 (Fig. 6d, e; Additional file 8: Figure S1e). Integrated with our observation on rapid growth for developing seeds at 10–30 DAF (Fig. 1b, c), we could infer that high transcriptional LTP1/4/6 may be involved in seed growth of *P. sibirica*.

## Discussion

In this study, a concurrent evaluation of growth tendency, oil content, FA compositions, biodiesel yield and fuel properties as well as model construction for fuel property prediction was conducted on developing seeds from *P. sibirica* plus tree (germplasm accession AS-80) (Tables 1 and 2; Fig. 1b–d), revealing that the oils of developing seeds harvested after 60 DAF may be as novel potential feedstock for producing biodiesel with ideal fuel properties. However, current knowledge of how carbon or energy is channeled to oil accumulation in oilseed plants is still limited. To better develop the seed oils of developing *P. sibirica* as woody biodiesel, we performed a global analysis of transporters in developing seeds of *P. sibirica* at transcript level based on our recent 454 sequencing data [49], resulting in 116 genes to annotate for membrane transporters in different organelles. By qRT-PCR and RT-PCR, some key transporters related to carbon allocation, precursor transport and energy supply were identified for FA synthesis, TAG assembly and oil mobilization, which allowed us to unreal the transporter-mediated mechanism of high oil production in developing *P. sibirica* seeds for better development of woody biodiesel.

### Transporters specifically for carbon allocation from sucrose to oil synthesis in developing seeds

In oilseed plants, photosynthetic sucrose stored in vacuole is transferred to sink organs as carbon source for oil synthesis via apoplastic pathway by sucrose transporter (SUT) [60–62]. Our finding of a close correlation between high transcriptional SUT2/4 and active oil synthesis of developing seeds (Figs. 1d, 3c; Additional file 8: Figure S1d) revealed an essentiality of SUT2/4 in sucrose import into cytosol as carbon source for FA synthesis in developing seeds of *P. sibirica*, as was noted in developing fruits of peach and pear [63, 64], which could support the idea of main carbon supply for fruit or seed as apoplastic sucrose [7, 17]. Given the fact of less transcript (Fig. 3c) for tonoplast transporters (VGT1 and TMT1) in relation to import hexose from cytosol into vacuolar [65], it could be speculated that differential transcripts between VGT1/TMT1 (down-regulation) and SUT2/4 (up-regulation) may contribute to provide cytosolic hexose for glycolysis during FA synthesis of developing *P. sibirica* seeds.

A major flux through glycolysis is expected to provide precursors responsible for high oil synthesis in oilseed plants [7, 11, 17, 20, 22, 30, 32, 66], all of which are needed to be imported into plastid by selective transporters. Our observations that both PPT1 (PEP transporter) and BASS2 (PYR carrier) showed high transcript and a positive correlation with active oil synthesis of developing seeds (Figs. 1d, 3a; Additional file 8: Figure S1a) revealed that the PPT1/BASS2-mediated unidirectional transport may contribute to provide PEP/PYR from cytosol into plastid glycolysis for FA synthesis destined to oil production in developing seeds of *P. sibirica*, which could be evidenced by the fact that overexpression of *BASS2* or *PPT1* increased oil content in Arabidopsis and tobacco seeds [67, 68]. In addition to glycolysis, FA synthesis was also fed by Rubisco shunt and OPPP [66, 69–72]. The participation of such pathway in our work could be clearly demonstrated by rich transcription for G6P transporter (GPT1), plastidial OPPP and Calvin cycle-related enzymes (SBP, RBCS1B, GAPA/B and PRK) at 10–30 DAF (Figs. 3a and 4a, b), implying that a portion of GPT1-mediated G6P import into plastid may be essential for FA synthesis in early developing seeds, as was the case for other oilseed plants [66, 70–72].

Altogether, abundant transcripts of transporters (SUT2/4, GPT1, PPT1 and BASS2) were correlated with the increases of oil content and biodiesel yield of developing seeds (Figs. 1d and 3a, c), revealing its importance in allocation carbon source (sucrose) for high oil synthesis and biodiesel yield during *P. sibirica* seed development.

### Transporters specifically involved in ATP provision for de novo FA synthesis in developing seeds

In addition to carbon supply, de novo FA synthesis in plastid requires ATP and reducing power, most of which are derived from other organelles by the transporters [20]. Here, high transcripts were identified for both plastidial NTT2 relevant to import cytosolic ATP in exchange with ADP [27–29] and mitochondria ACC3 as ATP exporter in exchange with cytosolic ADP [33, 34] in developing seeds (Fig. 3a, b; Additional file 8: Figure S1a, c), paralleled to the active oil synthesis (Fig. 1d), emphasizing that coordinated transcripts of NTT2/ACC3 may provide a strong channeling of ATP into plastid for FA synthesis of developing seeds. Evidence for this conclusion was the finding (Fig. 3a, b) that PIC1 as transporter of cytosol Pi into mitochondria [73] showed high transcript, but less transcript was noted for PHT2.1/4.2/4.5 relevant to Pi import into plastid [74], implying its key role in supply mitochondrial Pi for ATP synthesis during seed development. It seems, therefore, that these bidirectional transporter-mediated exchange of adenine nucleotides (ATP, ADP and AMP) and Pi among plastid, cytosol and mitochondria could help to generate ATP available for FA synthesis in plastid, destined for high oil yield, as also noted in Arabidopsis [34].

### Selective transport proteins involved in TAG synthesis destined for high-quality oil yield in developing seeds

Increasing TAG synthesis and oil yield would expand economic value for oilseed plants [37], but the mechanism of FA transport from plastid to ER for TAG assembly is still unknown. In recent years, some transport proteins of ABC and FAX have been shown for export plastid FA into ER for TAG synthesis [36, 37, 48, 75]. Of our identified 5 FAXs and 63 ABC transporters (Additional file 6: Table S6), only two plastid membrane-localized ABCB9/28 and FAX2 were highly expressed (Fig. 6b, c; Additional file 8: Figure S1e) and correlated with rapid oil synthesis (Fig. 1d) of developing seeds, so it was concluded that they may be as important transporters of FA export from plastid for TAG synthesis during seed development. This finding was contrasted with previous result of FAX1/ABCA9 as main supplier of plastid FA for TAG synthesis by both mutant and overexpression analyses [36, 48, 75]. We also noticed that ER-localized ACBP1, which in Arabidopsis transports acyl-CoA for TAG accumulation [44], increased transcript over the oil synthesis period of developing seeds (Figs. 1d, 6a), pointing to a role for ACBP1 as a key transporter of acyl-CoA substrate for ER TAG synthesis during seed development of *P. sibirica*.

Also of note was involvement of ACBP in control the partition of 18:1 between elongation and desaturation at ER [40]. Our identification of high transcript for cytosolic ACPB4/6 before 50 DAF (Fig. 6a), the period at which polyunsaturated FA was actively synthesized (Fig. 2a; Table 1), implying that ACPB4/6-mediated transport may be crucial for polyunsaturated FA accumulation in developing *P. sibirica* seeds, which was evidenced by polyunsaturated FA increase in *ACBP4/6*-overexpressing Arabidopsis seeds [45–47]. Our previous works have indicated that raw seed oils for high-quality biodiesel were dependent on the ideal contents of polyunsaturated (< 60%), monounsaturated (> 30%) and saturated FAs (< 30%) [50], which was also shown in this study that the contents of polyunsaturated (18.9–23.0%), monounsaturated (73.0–77.1%) and saturated FAs (4.0–4.1%) were detected at 60–70 DAF (Fig. 2a; Table 1), during which the developing seeds were identified with high-quality oil, high-yield biodiesel and superior fuel properties (Fig. 1d; Table 2). This, integrated with high transcript for FA/acyl-CoA transport-related proteins (ABCB9/28, ACBP1 and FAX2) during TAG synthesis (Fig. 6a–c), revealed that coordinated transcripts of ABCB9/28, FAX2 and ACBP1/4/6 may contribute to affect FA composition and oil yield, destined to the eventual biodiesel yield increase and fuel property improvement in developing seeds of *P. sibirica*.

### Transporter-mediated regulation for a limited loss of TAG breakdown during seed maturation

A decline in oil content during seed maturation has been reported in oilseed plants [76–78]. During storage oil mobilization, cytosolic FAs (from TAG lipolysis) and ATP are imported into peroxisome by the transporters of CTS and PNS for acyl-CoA formation and then degraded to acetyl-CoA via β-oxidation, which is further converted by glyoxylate cycle or TCA cycle [37, 38, 79]. Our findings that transcripts for PNC1 and CTS increased after 60 DAF, a temporal pattern also noted for TAGL2 and SDP1 (known for TAG breakdown) (Figs. 3d, 5a), revealed main contribution of them to TAG mobilization at seed maturity phase of *P. sibirica*. Also consistent with this conclusion, high transcript was characterized for mitochondria transporters of SFC and DIC1 (Fig. 3b) in relation to gluconeogenesis [35, 80], and enzymes of glyoxylate cycle (ACO3, CYS2, ICL and MLS), gluconeogenesis (PEPCK2) and β-oxidation (LACS6, MEP2 KAT2 and ACX2/3) after 60 DAF (Figs. 5b–d). Given that knockout of *pnc1*/*2*, *cts*, *sfc* or *dic1* promoted oil increase in mature seeds of transgenic plants [78, 81–86], together with our detection of only 2.1% decline of oil content for ripening seeds of *P. sibirica* (Fig. 1d), it was believed that the transporter-mediated TAG breakdown during seed maturity was limited, which could also be supported by lower transcript levels of transporters for TAG mobilization than for both FA synthesis and TAG assembly during seed development of *P. sibirica* (Figs. 3, 4, 5 and 6).

**Fig. 7:**
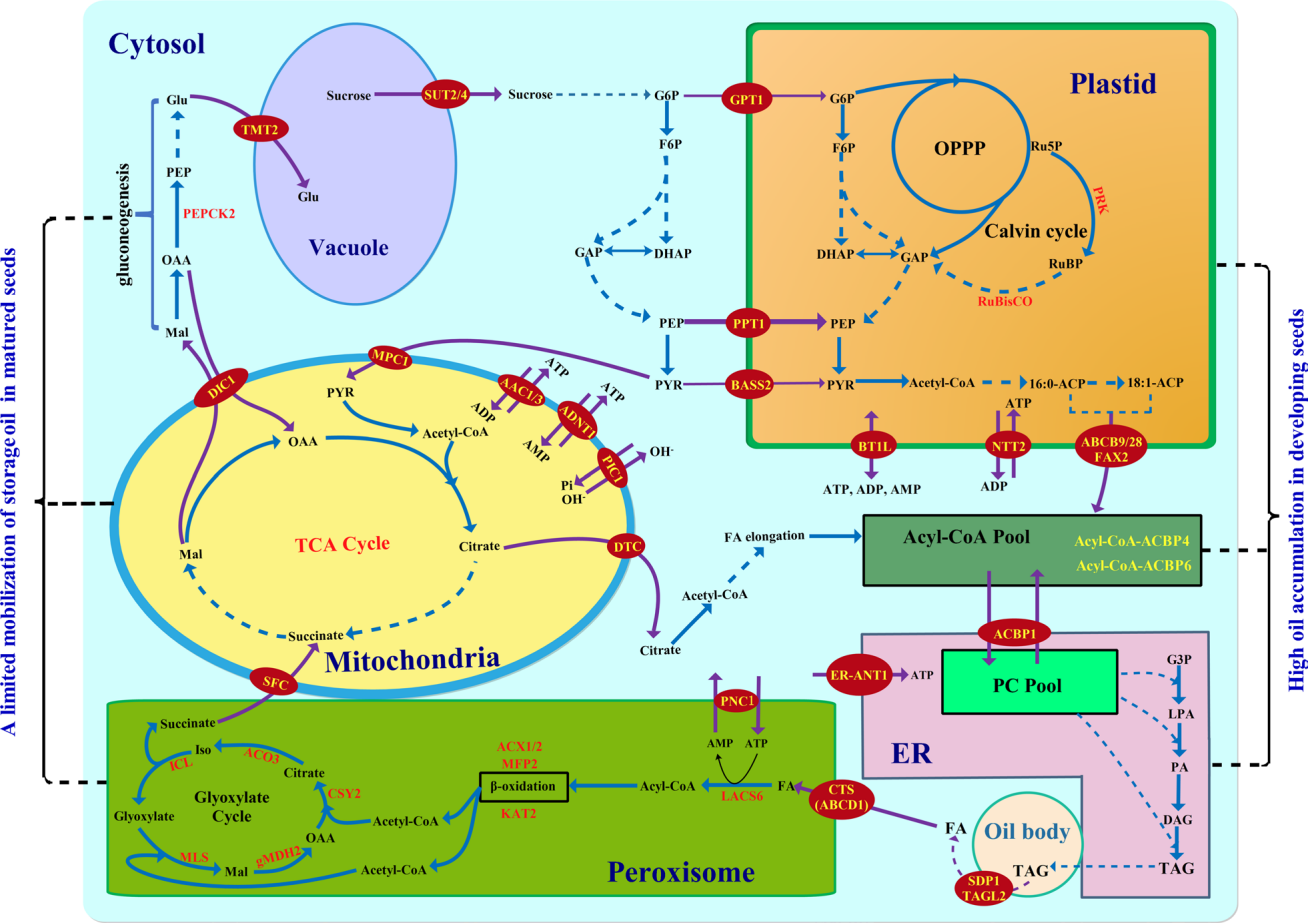
Characterization of complex transporter-mediated model of carbon allocation and energy supply for oil synthesis in developing *P. sibirica* seeds. The identified intracellular metabolite transport routes of carbon allocation and energy supply for oil synthesis are mainly based on a combination of our previous 454 sequencing data analysis and qRT-PCR detection. The background color distinguishes different subcellular locations and/or pathways as follows: Light green signifies a direct involvement in glyoxysomal glyoxylate cycle and β-oxidation; light blue signifies a cytosolic location; yellow signifies mitochondrial TCA cycle; orange signifies plastidial glycolysis and OPPP; light purple signifies the vacuole; pink signifies the ER membrane. Dark purple arrows represent the transports of metabolite and energy across intracellular membrane by specific transporters. All transporters involved in carbon flux allocation and energy provision for oil synthesis and TAG mobilization are shown in yellow. Abbreviations for the transporters, enzymes and metabolites are as follows: *AAC* ATP/ADP carrier, *ACBP* acyl-CoA-binding protein, *ACO* aconitase, *ACX* acyl-CoA oxidase, *ADNT* adenine nucleotide carrier, *AtUTr* nucleotide sugar transporter, *BASS* pyruvate (PYR) carrier (bile acid: sodium symporter family protein), *BOU* acetyl-carnitine/carnitine carrier, *BT1L* adenine nucleotide uniporter, *CN* cetane number, *CFPP* cold filter plugging point, *CP* cloud point, *CTS* COMATOSE, *CYS* citrate synthase, *D* density, *DIC* dicarboxylate carrier, *DTC* dicarboxylate/tricarboxylate carrier, *ER* endoplasmic reticulum, *ER-ANT1* ER membrane ATP transporter, *F6P* fructose-6-phosphate, *FAMEs* FA methyl esters, *FAX* FA exporter, *GAP* glyceraldehyde 3-phosphate, *GLT* glycolipid transporter, *GPT* glucose-6-phosphate (G6P) transporter, *ICL* isocitrate lyase, *IV* iodine value, *KAT* 3-ketoacyl-CoA thiolase, *KV* kinematic viscosity, *LACS* long-chain acyl-CoA synthase, *LTPs* lipid transfer proteins, *MLS* malate (Mal) synthase, *gMDH* glyoxysomal NAD-Mal dehydrogenase, *MPC1* PYR carrier, *NTT* nucleoside triphosphate (NTP) transporter, *OPPP* oxidative pentose phosphate pathway, *OS* oxidation stability, *PC* phosphatidylcholine, *PEPCK* phosphoenolpyruvate (PEP) carboxykinase, *PHT* phosphate (Pi) transporter, *PIC* Pi carriers, *PNC* peroxisomal adenine nucleotide carrier, *PPT* PEP transporter, *PRK* phosphoribulokinase, *RBCS* ribulose-1,5-bisphosphate carboxylase/oxygenase (Rubisco) small subunit, *RPE* ribulose-5-phosphate (Ru5P) epimerase, *RPI* ribose 5-phosphate isomerase, *SBP* sedoheptulose-bisphosphatase, *SDP1* sugar dependent 1, *SFC* succinate (SUC)/fumarate carrier, *SUT* sucrose transporter, *TA* transaldolase, *TAG* triacylglycerol, *TAGL* TAG lipase, *TCA* tricarboxylic acid, *TK* transketolase, *TMT* tonoplast monosaccharide transporter, *TPT* triose phosphate (TP) transporter, *VGT* vacuolar glucose transporter, *XPT* xylulose 5-phosphate (X5P)/phosphate transporter

Overall, effective transport of carbon source and energy into plastid for de novo FA synthesis, in conjunction with the transporter-mediated FA import into ER for TAG synthesis and a limited loss of oil mobilization (Fig. 7), may be crucial for the eventual high yield of oil and biodiesel in developing seeds of *P. sibirica*.

## Conclusions

In this work, to better develop the seed oils of *P. sibirica* as woody biodiesel, one plus tree (germplasm accession AS-80) with high-quantity seed oils was selected as experimental material and then a subsequent exploration of dynamic patterns for oil accumulation, growth tendency and the biodiesel yield and fuel properties as well as the construction of prediction model for fuel properties was made during seed development, revealing that the oils from developing seed harvested at 60 DAF may be as potential raw material suitable for producing biodiesel with optimum fuel properties. Such assessment could provide a pivotal reference for exploitation of woody biodiesel plants. In an attempt to highlight the transport mechanism for oil synthesis during *P. sibirica* seed development, a global analysis of transporter was performed in developing *P. sibirica* seeds based on our recent 454 sequencing data, and a total of 116 genes for membrane intrinsic transporters in mitochondria, plastid, vacuole, peroxisome and ER were annotated. The differential transcript analysis by RT-PCR and qRT-PCR has led to the identification of some key transporters in carbon allocation and energy supply as well as intermediate transport responsible for FA synthesis and TAG assembly during seed development, including mitochondria transporter (ACC1/3, ADNT1, PIC1, MPC1 and DTC), plastid transporter (BASS2, GPT1, PPT1, NTT2, FAX2, PHT2.1/4.2/4.5, ABCB9/28 and BT1L), ER transporter (ER-ANT1) and tonoplast transporter (SUT2/4). We also marked some transporters (PNC1, DIC1, CTS and SFC) for TAG breakdown during seed maturity. Together, our findings and complex transporter-mediated metabolic model (Fig. 7) will provide a better insight into regulatory mechanism of high oil production and may also provide both a rich source of data and of considerable interest to those studying woody biodiesel plants.

## Materials and methods

### Collection of plant materials

The *P. sibirica* seeds from different developmental stages were collected from 10-year-old plus tree (germplasm accession AS-80) planting in Changping District of Beijing (E116°23′, N40°22′), China. The flowers of twenty trees with the same anthesis were marked and then one hundred fruits (5 fruits per tree) were harvested at 10 (immature stage), 20, 30, 40, 50, 60 and 70 DAF (full mature stage), respectively. After removing the sarcocarp, fresh seeds were immediately frozen in liquid nitrogen and stored at − 80 °C until use for qRT-PCR and RT-PCR assay. The fresh weight of seeds was measured with an electronic balance and the longitudinal and transverse diameter of seeds was determined with vernier caliper. All the determination was conducted in triplicate.

### Seed oil extraction and trans-esterification

The fresh seeds from every developing stage were stored at room temperature for 1 week to dry until the samples attained uniform weight. About 30 g of dry seeds (3 samples per developing period) was crushed into an average particle size of 0.8 mm using domestic grinder and then the oils were extracted with petroleum ether using Soxhlet apparatus at 45–50 °C [14]. After extraction for 6–8 h, the oil was separated from organic mixture by rotary evaporator (LABORTA 4000-efficient, Germany) and then dried at 105 °C in ventilated drying oven. The content of extracted oils from each developing seeds was calculated as the difference between the weights of dry seed sample before and after extraction and the results were expressed as the percentage of the extracted oil weight to dry seed weight (%, g/g). To analyze FA compositions, the oils from developing seeds were *trans*-esterified as previously described method [50]. All analysis was performed in triplicate.

### FA methyl ester analysis and biodiesel yield calculation

The FA methyl esters (FAMEs) obtained from each developmental seeds was analyzed for determining the FA compositions using an Agilent 6890 (California, USA) gas chromatograph equipped with a flame ionization detector (GC-FID) as previously described [5]. The HP-INNOWax capillary column (inner diameter 0.32 mm, filmthickness 0.5 μm, split 1:20) was used and the temperature was programmed at 60 °C, with a rise of 4 °C min^−1^ to 220 °C and heated to 240 °C for 10 min. The carrier gas was helium with a flow rate of 1.0 mL min^−1^. The peaks of FAMEs were identified by comparing their retention time with that of the known standards, and peak integration was performed by applying HP3398A software. Each FAME analysis was run in triplicate and the data were presented as the mean. The biodiesel yield was calculated by the previous method [8], where the yield was expressed as the percentage (%, g/g) of the obtained total amount of FAMEs (g) to the used amount of raw oils (g).

### Evaluation of biodiesel properties

The biodiesel fuel properties (IV, CN, CFPP, CP, OS, D and KV) were calculated from the FAME compositions according to the previously method [14, 50]. The determination was conducted in triplicate. The biodiesel fuel properties of seed oils were compared with the relevant specifications of EN 14214-2008 (European, 2008), ASTM D6751-2010 (USA, 2010), DIN V51606-1997 (Germany, 1997) and GB/T 20828-2007 (China, 2007).

### Construction of prediction model for biodiesel fuel properties of raw seed oils

The triangular prediction model of seed oils from different developing stages was constructed according to the influence of the FAME compositions [7, 50]. To predict the biodiesel properties of oils from developing seeds, the percentages of saturated, monounsaturated and polyunsaturated FAMEs from different developing seeds were calculated to outline a triangular prediction graph, in which three angular points of the triangle meant the 100% of monounsaturated, polyunsaturated and saturated FAMEs, respectively. In triangular graph, the region existed at the far end of the polyunsaturated angular point (lower left vertex) and the saturated angular point (lower right vertex) was delineated to predict the biodiesel fuel properties, taking into account the CN, IV, CFPP and OS [7, 50].

### Functional annotation of unigenes for the transporters in developing seeds

Based on our previous 454 pyrosequencing data (SRX339392) from different tissues of Siberian apricot [49], the unigenes were annotated to be involved in the transporters using BLASTX alignment against known protein databases of NCBI nonredundant, Arabidopsis proteome, SWISS-PROT, TREMBL, AP, CDD, PFAM and COG. Also, GO functional classifications were analyzed by GO terms (http://www.geneontology.org) using Blast2Go software, and KEGG pathway assignment was performed using the BLAST all against Kyoto Encyclopedia of Genes and Genomes database.

### Gene expression analysis

The total RNA was extracted using RNeasy Plant Mini Kits (Qiagen, Inc., USA) and the obtained RNA was qualified and quantified using Nanodrop ND-1000 Spectrophotometer (N Wilmington, DE, USA). All the samples showed a 260/280 nm ratio from 1.9 to 2.1 and then was reverse transcribed using the Reverse Transcription System (Promega). The qRT-PCR was conducted on 7500 Real-Time PCR System using SYBR Premix Ex Taq Kit (TaKaRa) based on the manufacturer’s protocol. All the amplified primers (Additional file 10: Table S9) were designed by PrimerQuest (http://www.idtdna.com/PrimerQuest/Home/Index) software with melting temperatures at 62 °C, and the absence of secondary structures was verified by UNAFold program (http://eu.idtdna.com/UNAFold). The genes encoding for cyclophilin (CYP) and ubiquitin-conjugating enzyme (UBC) were used as inner references as our previously described [87]. Negative controls consisting of nuclease-free water instead of template and reverse transcriptase controls prepared by substituting reverse transcriptase for nuclease-free water in cDNA synthesis step were included in all analyses for each primer pair. Additionally, the RT-PCR assay was conducted according to our previous study [87] and all gene-specific PCR products were confirmed by gel electrophoresis on 1.5% agarose gel and visualized after staining with ethidium bromide using the Gene Genius Imaging System and quantified with GeneTool software (Gene Company Limited). Three biological replicates with three technical repetitions each were performed for qRT-PCR and RT-PCR.

## Additional files

**Additional file 1: Table S1.** Annotation information of unigenes for *P. sibirica* by BLAST searches against public databases. They mainly involved in carbon allocation and energy provision (glycolysis, TCA cycle, ATP synthesis, OPPP and FA β-oxidation), the transports of metabolite (sucrose, precursor, adenine nucleotide, FA, acyl-CoA and lipid) for FA synthesis, TAG assembly and oil mobilization as well as enzymes involved in FA synthesis and TAG assembly during seed development.

**Additional file 2: Table S2.** Genes annotated for plastidial transporters and OPPP in developing *P. sibirica* seeds. Plastidial transporters involved specifically in carbon allocation into glycolysis and OPPP, and the transport of adenine nucleotide and Pi. Also, some genes were annotated for enzymes of OPPP in both plastid and cytosol.

**Additional file 3: Table S3.** Genes annotated for mitochondrial metabolite transporters in developing *P. sibirica* seeds. They mainly involved in ATP synthesis, TCA cycle and oil mobilization.

**Additional file 4: Table S4.** Genes annotated for metabolite transporters of tonoplast in developing *P. sibirica* seeds. They mainly involved in the transports of sugar, malate and sugar alcohol.

**Additional file 5: Table S5.** Genes annotated for transporters of ER membrane in developing *P. sibirica* seeds. They included ATP transporter (ER-ANT1) and nucleotide sugar transporter (AtUTr1).

**Additional file 6: Table S6.** Genes annotated for other transport proteins for FA/lipid transport in developing *P. sibirica* seeds. They mainly included ATP-binding cassette (ABC) proteins, acyl-CoA-binding proteins (ACBPs), lipid transfer proteins (LTPs) and trigalactosyldiacylglycerol 1 (TGD1) protein.

**Additional file 7: Table S7.** Genes annotated for transporters and enzymes for TAG mobilization in developing *P. sibirica* seeds. These genes included TAG hydrolysis, peroxisomal transporter, FA β-oxidation, glyoxylate cycle and gluconeogenesis.

**Additional file 8: Figure S1.** Expression analysis of transporters at various organelles in developing *P. sibirica* seeds by RT-PCR. (a) Plastidial transporters. (b) Transporters of peroxisomal and ER membrane. (c) Mitochondrial transporters. (d) Metabolite transporters of tonoplast. (e) Other key transport proteins. The gene encoding for ubiquitin-conjugating enzyme (UBC) was used as internal control.

**Additional file 9: Table S8.** Genes annotated for enzymes of Calvin cycle in developing *P. sibirica* seeds.

**Additional file 10: Table S9.** The information of all primers used in this study for qRT-PCR and RT-PCR analysis.

## Abbreviations

AAC: ATP/ADP carrier
ACBP: acyl-CoA-binding protein
ACO: aconitase
ACX: acyl-CoA oxidase
ADNT: adenine nucleotide carrier
AtUTr: nucleotide sugar transporter
BASS: pyruvate (PYR) carrier (bile acid: sodium symporter family protein)
BOU: acetyl-carnitine/carnitine carrier
BT1L: adenine nucleotide uniporter
CN: cetane number
CFPP: cold filter plugging point
CP: cloud point
CTS: COMATOSE
CYS: citrate synthase
D: density
DAF: days after flowering
DIC: dicarboxylate carrier
DTC: dicarboxylate/tricarboxylate carrier
ER: endoplasmic reticulum
ER-ANT1: ER membrane ATP transporter
F6P: fructose-6-phosphate
FAMEs: FA methyl esters
FAX: FA exporter
GAP: glyceraldehyde 3-phosphate
GLT: glycolipid transporter
GPT: glucose-6-phosphate (G6P) transporter
ICL: isocitrate lyase
IV: iodine value
KAT: 3-ketoacyl-CoA thiolase
KV: kinematic viscosity
LACS: long-chain acyl-CoA synthase
LTPs: lipid transfer proteins
MLS: malate (Mal) synthase
gMDH: glyoxysomal NAD-Mal dehydrogenase
MPC1: PYR carrier
NTT: nucleoside triphosphate (NTP) transporter
OPPP: oxidative pentose phosphate pathway
OS: oxidation stability
PEPCK: phosphoenolpyruvate (PEP) carboxykinase
PHT: phosphate (Pi) transporter
PIC: Pi carriers
PNC: peroxisomal adenine nucleotide carrier
PPT: PEP transporter
PRK: phosphoribulokinase
PYR: pyruvate
RBCS: ribulose-1,5-bisphosphate carboxylase/oxygenase (Rubisco) small subunit
RPE: ribulose-5-phosphate (Ru5P) epimerase
qRT-PCR: quantitative real-time RT-PCR
RT-PCR: semi-quantitative RT-PCR
SBP: sedoheptulose-bisphosphatase
SDP1: sugar dependent 1
SFC: succinate (SUC)/fumarate carrier
SUT: sucrose transporter
TA: transaldolase
TAG: triacylglycerol
TAGL: TAG lipase
TCA: tricarboxylic acid
TMT: tonoplast monosaccharide transporter
TPT: triose phosphate (TP) transporter
VGT: vacuolar glucose transporter
XPT: xylulose 5-phosphate (X5P)/phosphate transporter
