# Supplementary figures and images for: Comprehensive evaluation of fuel properties and complex regulation of intracellular transporters for high oil production in developing seeds of Prunus sibirica for woody biodiesel
Source: Biotechnol Biofuels. 2019 Jan 4;12:6. doi: 10.1186/s13068-018-1347-x (PMC6318995; doi:10.1186/s13068-018-1347-x)

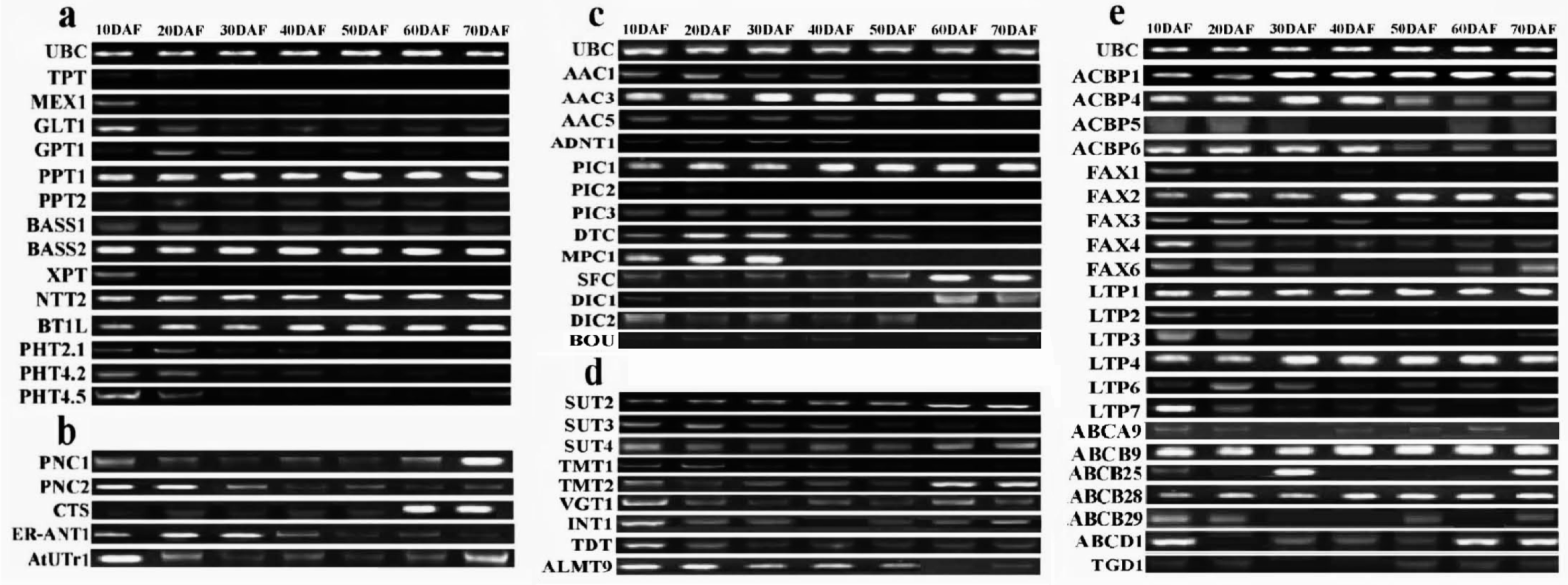

Supplement: Supplementary file 8 — Additional file 8: Figure S1. Expression analysis of transporters at various organelles in developing P. sibirica seeds by RT-PCR. (a) Plastidial transporters. (b) Transporters of peroxisomal and ER membrane. (c) Mitochondrial transporters. (d) Metabolite transporters of tonoplast. (e) Other key transport proteins. The gene encoding for ubiquitin-conjugating enzyme (UBC) was used as internal control. [file 13068_2018_1347_MOESM8_ESM.tiff]
